# Case report: Visibly curative effect of dabrafenib and trametinib on advanced thyroid carcinoma in 2 patients

**DOI:** 10.3389/fonc.2022.1099268

**Published:** 2023-01-05

**Authors:** Xue Peng, Jianyong Lei, Zhihui Li, Kun Zhang

**Affiliations:** Thyroid and Parathyroid Surgery Center, West China Hospital of Sichuan University, Chengdu, China

**Keywords:** dabrafenib, trametinib, lymph node metastasis, thyroid carcinoma, targeted drug

## Abstract

**Background:**

Differentiated thyroid cancer accounts for the majority of thyroid cancers and has a good prognosis after standard treatment. However, there are still some complex and refractory thyroid cancers, including locally advanced differentiated thyroid carcinoma and medullary carcinoma (MTC), poorly differentiated thyroid carcinoma (PDTC), and anaplastic thyroid carcinoma (ATC). Here, we report the therapeutic response of 2 advanced thyroid carcinoma patients treated with dabrafenib and trametinib.

**Case presentation:**

Two elderly females presented to the clinic with neck masses, dyspnea, and dysphagia. Signs of the trachea and esophageal compression were markedly visible in computed tomography (CT) scan and ultrasonography. Pathologic diagnoses of PDTC were confirmed for both patients through ultrasound-guided fine-needle aspiration (US-FNA). Both patients were significantly relieved from dyspnea and dysphagia after a course of treatment with dabrafenib and trametinib, and their tumors gradually shrank during the follow-up period.

**Conclusion:**

Overall, this treatment modality is rare, but effective. By sharing these 2 case reports, we hope to provide a reference for the treatment of clinically similar patients with advanced thyroid carcinoma.

## Introduction

Locally advanced thyroid carcinoma is a type of thyroid cancer in which the tumor markedly invades surrounding organs and structures. Especially, poorly differentiated thyroid carcinoma is more aggressive and rapidly progresses to distant metastases. Poorly differentiated thyroid carcinoma is an aggressive form of follicular cell-derived thyroid carcinoma with a behavior, morphology, and prognosis that is intermediate between indolent differentiated thyroid carcinoma (DTC) and rapidly growing and often fatal ATC (1). PDTC is a rare disease that was first described by Sakamoto in 1938 (2) and is reported to occur in 2% to 15% of all thyroid carcinomas (1, 3). Starting in 2004, the World Health Organization (WHO) classified PDTC as a non-follicular non-papillary, thyroglobulin-producing thyroid carcinoma with intermediate behavior between well-differentiated and anaplastic carcinoma (4–6). The 5-year disease-specific survival rate (DSS) for PDTC patients (51%) is also intermediate between DTC (91%) and ATC (0%) patients (7). PDTC patients usually miss the opportunity for surgery or have poor surgical outcomes due to the cancer’s invasiveness and quick progression to distant metastasis. PDTC does not have a standard of treatment internationally (8). Surgery, chemotherapy, and radioiodine therapy (RAI) are commonly used treatment schemes, but the treatment options for PDTC patients with advanced, critical illness are very limited (9). In this case report, we describe 2 patients with advanced thyroid carcinoma who received dabrafenib and trametinib with good outcomes.

This study was reported in agreement with principles of the CARE guidelines (10).

## Case presentation

### Case 1

In December 2021, a 72-year-old woman with a neck mass, dysphagia with a cough and expectoration was referred to the Department of Thyroid Surgery & Western Medicine, West China Hospital, Sichuan University. Before visiting our hospital, the patient found a hard mass on the left side of her neck, along with tenderness and poor mobility. At that time, there was no dyspnea or dysphagia. The patient was treated at Sichuan Cancer Hospital, underwent ultrasound examination and received FNA. Ultrasound showed morphological abnormalities in the thyroid, abnormal lymph nodes were found on both sides of the neck, and FNA cytology revealed PTC (occupying the right lobe of the thyroid). The results of immunohistochemical analysis were as follows: positive TTF-1, G-3, CK19, MC (HBME-1), CD56, Ki67 (10%), negative (TG), PD-L1: 22c3, CPS: 65, negative control: negative, and positive control: positive. Gene testing revealed a *BRAF V600E* gene mutation (Ct value was 20.16). Because the patient’s nutritional status was poor, and she could not afford immunotherapy, she was discharged from the hospital and took 3 cycles of anlotinib 12 mg qd (taking the medication for 2 weeks and stopping for 1 week). After completing the 3 cycles, the patient had dysphagia, increased coughing, and expectoration accompanied by blood in the sputum, so she was re-admitted to Sichuan Cancer Hospital. During hospitalization, the patient was unable to eat and was completely supported by parenteral nutrition.

**Figure 1:**
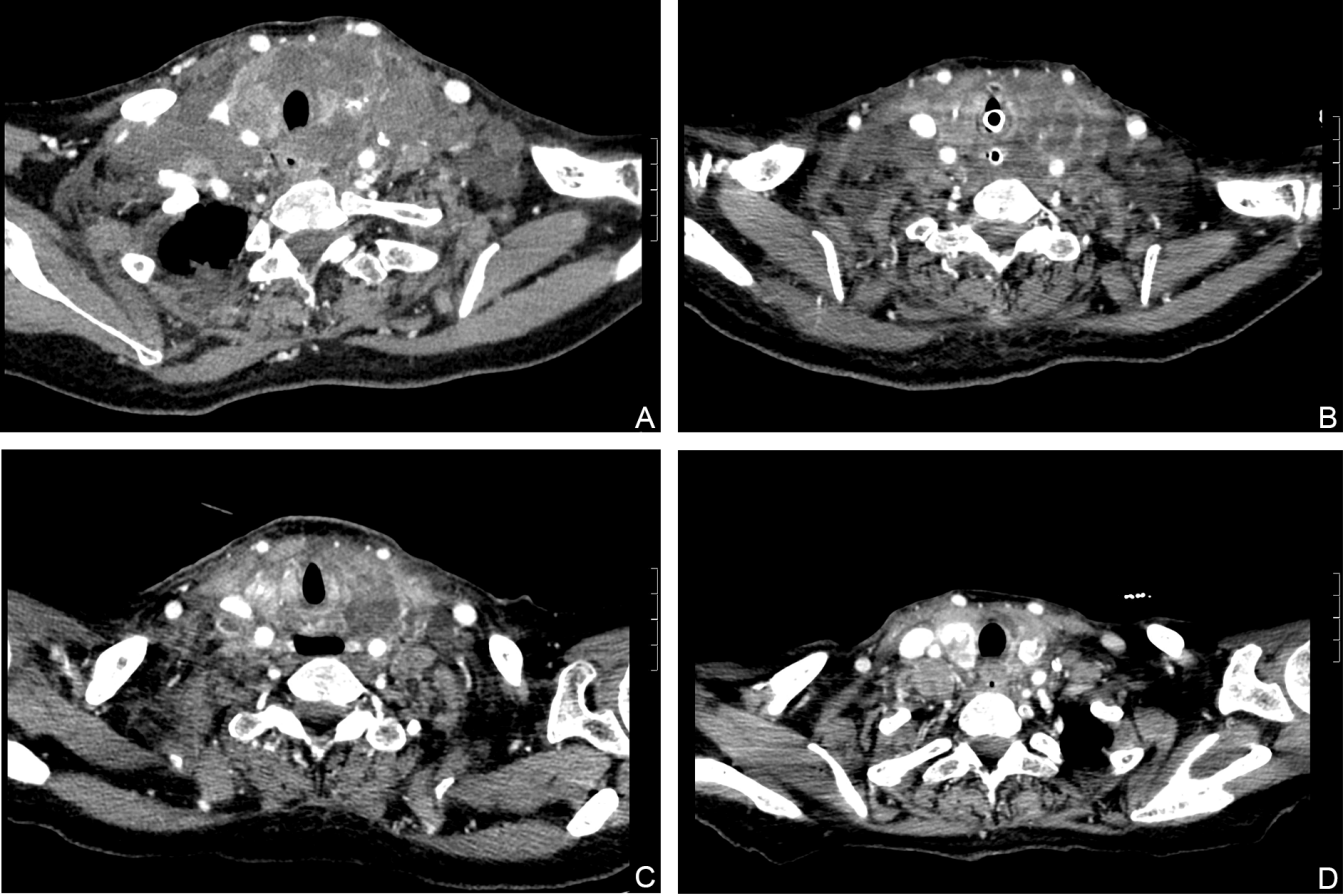
Hematoxylin and eosin (HE) stained FNAB of thyroid lobe **(A)**, 20× magnification) revealed malignant epithelial cells. IHC staining showed that malignant cells in thyroid were positive for G-3 **(B)**, 20× magnification), and negative for TTF1 **(C)**, 20× magnification), TG **(D)**, 20× magnification).

Physical examination showed that she had a hoarse voice, clear consciousness and diffuse swelling of the neck, and was unable to touch her trachea because of the trachea was compressed by a huge mass. The thyroid mass was hard, and its size could not be measured. The laboratory examination revealed the following: FT3 1.64 pmol/L (reference range, 3.60–7.50 pmol/L), FT4 9.53 pmol/L (reference range, 12.0–22.0 pmol/L), TSH 7.34 mU/L (reference range, 0.27–4.20 mU/L), thyroglobulin (hTg) < 0.04 µg/l (reference range, 3.5–77 µg/l), anti-thyroglobulin antibodies (TGAb) > 4000 IU/ml (reference range, 0–115IU/ml), and tumor marker/carcinoembryonic antigen (CEA) 0.64 ng/ml (reference range, 0–5 ng/ml). Ultrasound re-examination revealed that the thyroid gland contained giant nodules encircling the bilateral common carotid arteries. In addition, bilateral neck areas I-IV had multiple lymph node fusions, the left neck was approximately 12×10×16 mm, the right side was approximately 20×19×14 mm, and the corticomedullary demarcation was unclear. We showed on computed tomography (CT) that the thyroid gland was unclear and there were lymph node metastases in the neck and upper mediastinum (**Figure 1** **)**. The patient had a personal history of 20 years of hypothyroidism and had taken 50 µg levothyroxine (Euthyrox) for a long time. There was no history of neck surgery, neck radiotherapy, parathyroid disease, or hereditary disease.

**Figure 2:**
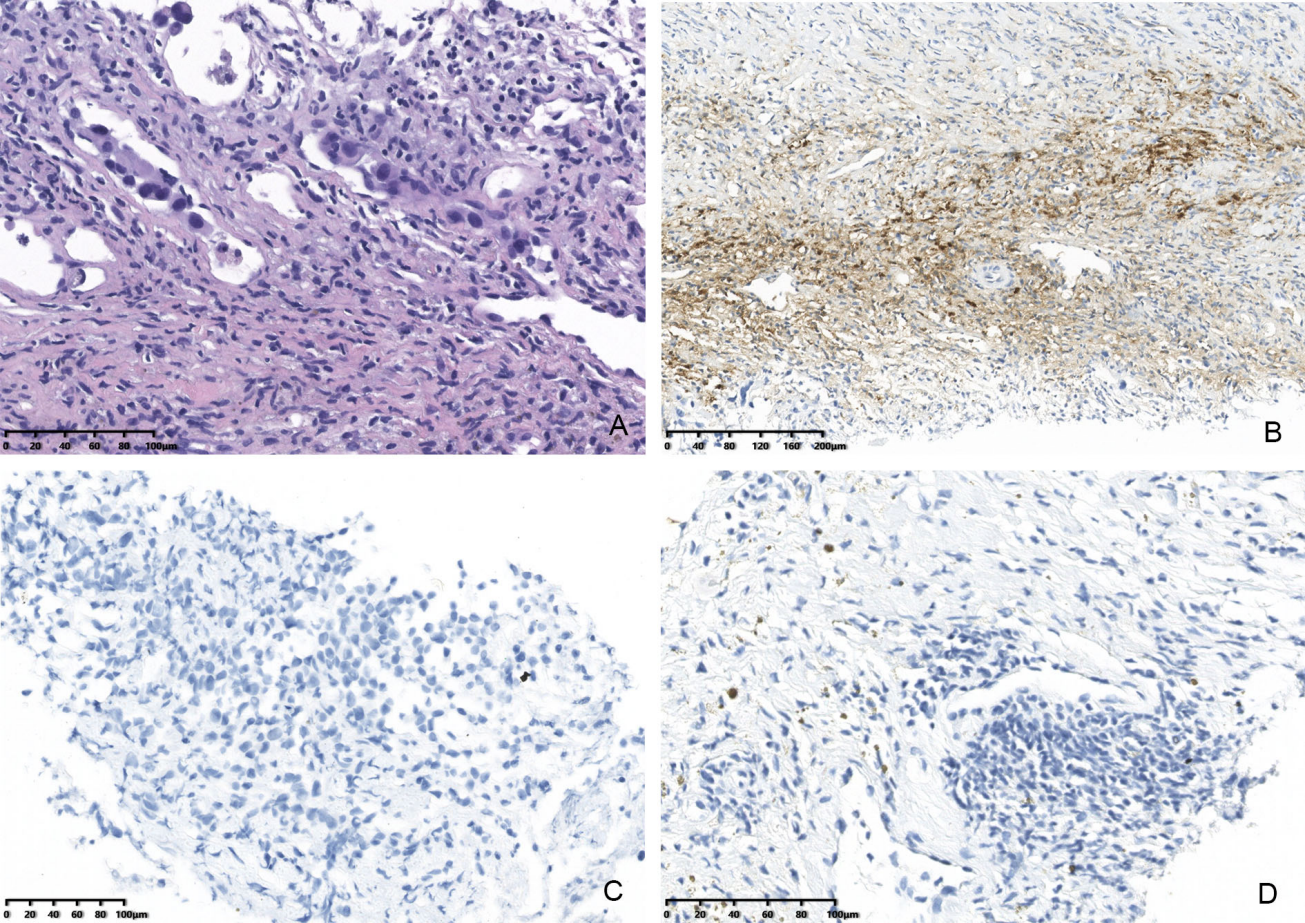
Computed tomography (CT) images showing that the size of the lesions had decreased. **(A)** Before treatment with dabrafenib and trametinib. **(B)** Dabrafenib and trametinib for 5 days. **(C)** Dabrafenib and trametinib for 19days. **(D)** Dabrafenib and trametinib for 50days.

To solve the patient’s nutritional problems, the endoscopy center placed a jejunal nutrition tube. During this process, the patient had sudden dyspnea with a sharp drop in oxygen saturation and partial pressure of carbon dioxide of 164%, pH: 6.9. The anesthesiology department performed a successful emergency nasotracheal intubation, and the patient was transferred to the critical care medicine department. The multidisciplinary team (MDT) ruled out surgical treatment, because of the high risks involved, and because the tumor was poorly demarcated from surrounding tissues. dabrafenib and trametinib treatment was finally considered. dabrafenib and trametinib is approved by the FDA for the treatment of patients with ATC and *BRAF V600E* mutations. So, we obtained consent from the patient’s family to tube feed the targeted agent dabrafenib mesylate (75 mg q12h) and trametinib (2 mg qd) on December 30, 2021. FDA recommends 150mg twice daily for the use of dabrafenib, but We had never used this scheme before in these two patients, so we could not evaluate the effectiveness and side effects. In addition, the patient’s condition was bad and the basic condition was poor, so we tried to use half a dose to observe the efficacy, and subsequently adjusted the scheme according to the patient’s condition. We sent the biopsy tissue for pathological re-examination, and the results of the cytology revealed malignant epithelial cells. Immunohistochemical staining of thyroid (**Figure 2** **)**, which exhibited positive CK19, CD56 and G-3, and negative TTF-1, TG, HBME-1, CGA, SYN, p63, CD20. The Ki-67 positive rate was approximately 40%. Confirmed poorly differentiated thyroid carcinoma. After 13 days of treatment, the patient’s dyspnea and dysphagia symptoms had decreased, and she could sleep in a flat position and drink a small amount of liquid. Her neck circumference gradually decreased with daily measurements. However, she still had hoarseness and developed black stool (approximately 100-200 g/d) and hematuria on a daily basis. According to the patient’s condition, the patient’s above symptoms improved after treatment with proton pump inhibitor (PPI), somatostatin and oral Yunnan Baiyao. To clarify the cause of bleeding would require a gastroscope, after the patient’s condition has improved. The patient continued oral dabrafenib 75 mg and trametinib 2 mg maintenance therapy and underwent thyroid stimulating hormone (TSH) repression therapy with levothyroxine 50μg after discharge. We compared the imaging reports at 3 days, 19days, 50days and found that the tumors significantly decreased in size. The author’s department subsequently found that the tumor did not continue to grow or have any detectable distant metastasis through outpatient service and telephone. At the end of February 2022, the patient experienced fever and seizure. Moreover, a new mass was found in the right neck. After discontinuing dabrafenib and trametinib for 10 days, the mass grew rapidly, and blood culture suggested a serious infection. Therefore, after comprehensive consideration, we recommended that she continue to take dabrafenib and trametinib. The patient’s condition progressed rapidly, and she died on March 10.

### Case 2

**Figure 3:**
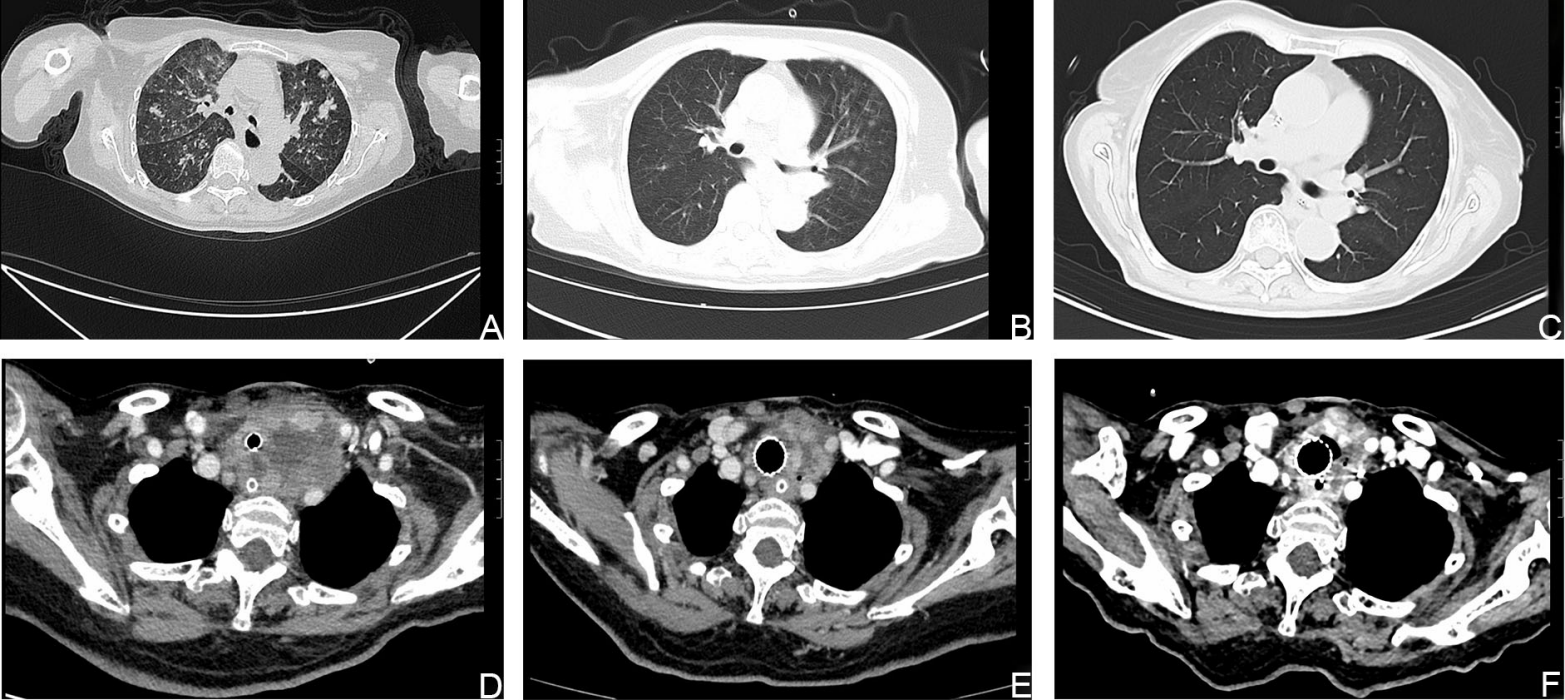
Computed tomography (CT) images showing that the size of the lesions had decreased. **(A, D)**. CT images of the metastatic lesions in the lung and neck respectively, before treatment with dabrafenib and trametinib. **(B, C, E, F)**. CT images of the metastatic lesions after treatment with dabrafenib and trametinib.

**Figure 4:**
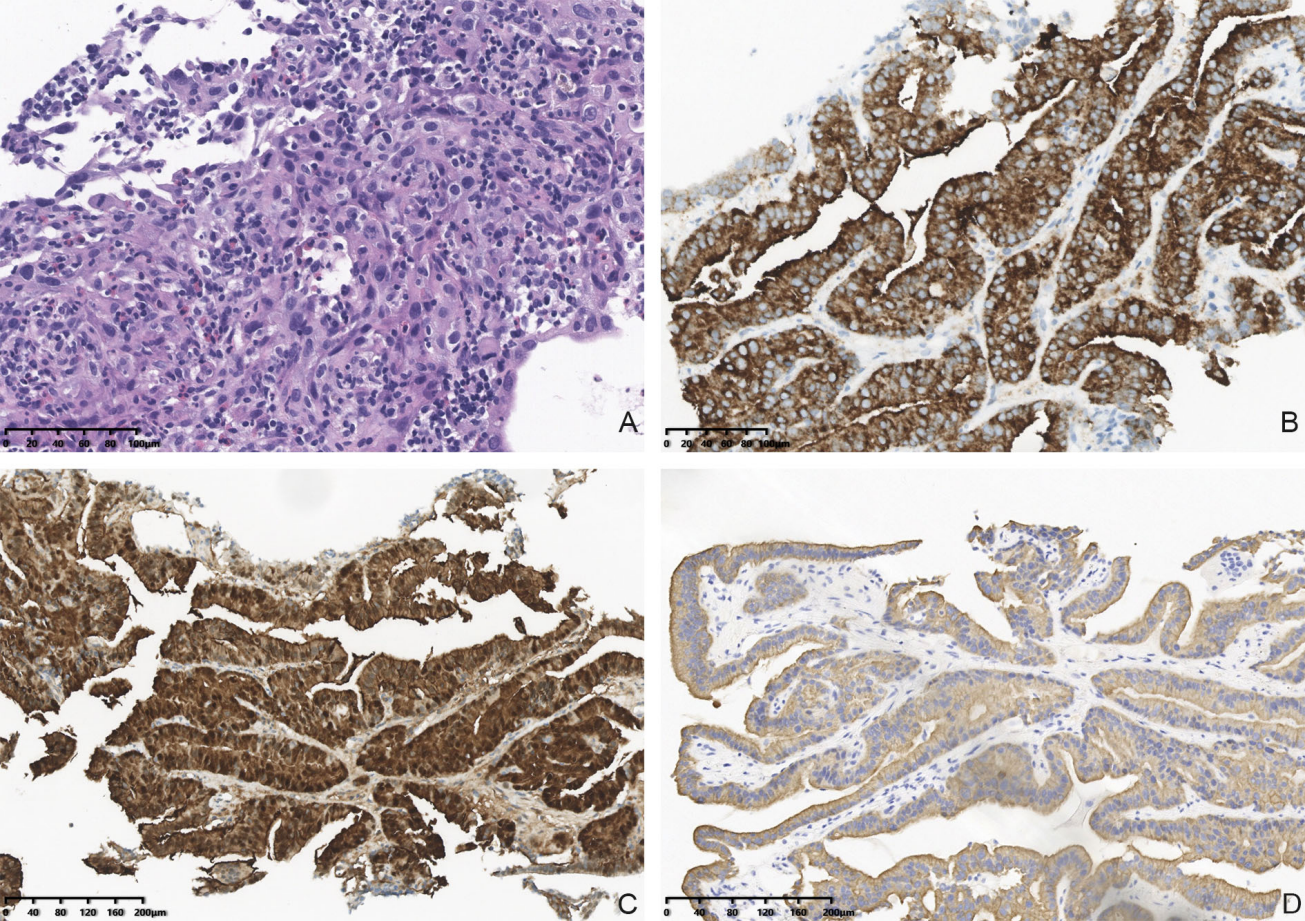
Hematoxylin and eosin (HE) stained FNAB of thyroid lobe **(A)**, 20× magnification) revealed malignant epithelial cells. IHC staining showed that malignant cells in thyroid were positive for BRAR-VE **(B)**, 20× magnification) and G-3 **(C)**, 20× magnification) and CK19 **(D)**, 20× magnification).

A 73-year-old woman presented to the emergency department of West China Hospital, Sichuan University, on January 31, 2022, because of a neck mass with chest and airway tightness. Previously, FNA was performed at her local hospital, and the results showed metastatic, poorly differentiated squamous cell carcinoma. Upon arrival in the emergency room, the patient suddenly lost consciousness. The emergency physicians immediately diagnosed respiratory failure and intubated the patient. To comprehensively evaluate the condition of patient, CT was performed and showed that there was a mass (6.3×5.6 cm) located in the left lobe region of the thyroid gland, which wrapped and invaded the trachea and esophagus, both of which were compressed to varying degrees, resulting in stenosis. Nodules of varying size were scattered throughout both lungs, with the largest nodule measuring 1.8 cm in diameter, which we suspect to be metastatic lesions **(** **Figure 3** **)**. The laboratory examination upon admission revealed the following: FT3 2.08 pmol/L (reference range, 3.60–7.50 pmol/L), FT4 9.39 pmol/L (reference range, 12.0–22.0 pmol/L), TRAb 2.79 IU/l (reference range, <1.75 IU/l), WBC 12.99×10/L (reference range, 3.5–9.5 10^9/L), Hb 99 g/L (115–150 g/L), and PLT 259×10/L (100–300 10^9/L); TSH, hTg, TGAb, and CEA were unremarkable. She had a 13-year history of papillary thyroid cancer (PTC) and had previously undergone total thyroidectomy, double central lymph node dissection, plus left cervical lymph node dissection in our hospital. Levothyroxine 75 µg in parallel with radioactive iodine [(131)I] therapy three times, were administered in after the operation. There was no personal or hereditary family history of cardiovascular or cerebrovascular disease. An appointment was set with a thyroid surgeon from our institution on the same day. After the completely treating the infection that emerged in the patient’s lungs during the consultation on February 7, 2022, she was admitted to the Thyroid and Parathyroid Surgery Center. A covered stent was used instead of tracheal intubation with the assistance of the Department of Otorhinolaryngology. Due to the patient was critically ill after admission and had obvious symptoms of neck compression. In an effort to strive for treatment time, while the patient underwent US-FNA, we communicated with the family and obtained the consent to empirically administer treatment with dabrafenib and trametinib (given the close similarity to that of case 1), and the final pathological results and genetic testing also confirmed the effectiveness of our medication. We selected a mass in the left lobe region of the thyroid gland by ultrasonographic localization, punctured the fine-needle into the interior of the nodule and multipoint aspiration was performed under the monitoring of ultrasound. Histological examination was performed postoperatively on fixed specimens with alcohol. Immunohistochemical analyses showed tissue positivity for the following markers: HBME-1, CK19, G-3, BRAF-VE, PCK, and PAX8. In addition, *TERT* promoter mutation (C288T) were identified by Molecular testing in the mass. Combined the morphology and all of the above findings led to a diagnosis of PTC with a mutation at position 228 of the *TERT* gene promoter **(** **Figure 4** **)**. Under receiving comprehensive treatment from the nutrition, infection, and respiratory departments, the patient’s condition improved after 5 days of dabrafenib and trametinib, and her symptoms of dyspnea, dysphagia, and hoarseness disappeared. Unexpectedly, she was required dose reductions of dabrafenib and adds radiotherapy because of edema of both lower extremities with petechiae. Three months later, the patient developed a tracheoesophageal fistula, which improved after conservative treatment. After ∼8 months of treatment, numerous metastatic lesions had lessened on computed tomography. The survival time of the patient has reached 10 months since she diagnosis of PTC. At present, the patient is in a state of no recurrence and metastasis, and there is no obvious adverse reaction after taking dabrafenib and trametinib.

Both were approved by the ethics committee of the West China Hospital, Sichuan University, and written informed consent was provided by the patient for publication of this case publication.

## Discussion

These cases give us great confidence and a new strategy for the clinical treatment of a advanced thyroid carcinoma. Our discussion will focus on the effects of treatment.

Targeted drugs are emerging as a new option for the treatment of a variety of advanced tumors in the clinic, raising the hope of survival for many patients with refractory tumors. Thyroid cancer-targeted drugs have been gradually introduced to the clinic in recent years, and better efficacy is also seen in many reports for the treatment of advanced thyroid carcinoma. We report encouraging results in 2 consecutive patients with severe comorbidities who were admitted to the Thyroid and Parathyroid Surgery Center, West China Hospital of Sichuan University. Both patients presented with life-threatening metastatic thyroid cancer and were not eligible for surgery or radiotherapy. Dabrafenib, a mutant BRAF kinase inhibitor, emerged from Glaxo Smith K’s research program for the discovery of selective inhibitors of mutant *BRAF* kinase activity for the treatment of solid tumors (11). Trametinib is an orally administered, reversible selective allosteric inhibitor of MEK1 and MEK2, and is also approved as a single agent in the treatment of melanoma (12, 13). dabrafenib and trametinib was first approved by the FDA on May 29, 2013, for the treatment of unresectable or metastatic melanoma with *BRAF V600E* or *V600K* gene mutations. After confirmation by clinical studies (14), the FDA approved dabrafenib and trametinib for patients with ATC and *BRAF V600E* mutations on May 4, 2018 (14, 15). The effect of the combination of the 2 drugs had only been observed in the treatment of ATC, but we cannot estimate whether the application of these two drugs to other types of thyroid carcinoma will also achieve satisfactory results. We tried this approach to treat the 2 patients with advanced thyroid cancer and BRAF mutation, and the effect was better than expected. White PS (16) reported 2 cases of metastatic papillary thyroid carcinoma with major complications. Metastases decreased, and thyroglobulin antibodies consistently decreased after treatment with dabrafenib and trametinib. The protocol adopted by the authors was treated initially with dabrafenib 150 mg twice daily plus trametinib 2 mg once daily, first in continuous daily dosing, then in a five-week-on and three-week-off schedule. Whereas our regimen was continuous use of dabrafenib 150 mg and trametinib 2 mg once daily. As with White PS, both patients showed rapid clinical improvement upon starting the regimen. Since there is no consensus on the combination of these two drugs for the treatment of non-ATC advanced thyroid cancer, we speculate that it may also achieve good efficacy for advanced thyroid carcinoma without ATC or *BRAF V600E* mutation. Although we do not have enough evidence to support that this regimen is available, and see few relevant reports. The experiences in the 2 patients presented here illustrate that administering the 2 drugs are feasible, and suggest that this approach is efficacious, even in cases with serious comorbidities. However, few data are currently available on drug efficacy and/or response, and further preclinical and clinical studies are needed.

According to the relevant studies and the drug instructions of dabrafenib, dabrafenib and trametinib is known to cause the following adverse events: fever, chills, skin toxicity, arthralgia, myalgia, cough, hypophosphatemia, hyponatremia, hyperglycemia, increased alkaline phosphatase, bleeding (defined as bleeding from a critical site or organ), etc (17). In case 1, the patient had black stool and hematuria on a daily basis, denied any history of gastrointestinal and urinary diseases, and denied NSAID drug use. Case 2 patient developed edema of both lower extremities with petechiae. We consider these phenomena to be a side effect. Some studies also report retinopathy, renal failure, fatigue, and rhabdomyolysis (17). The symptoms may largely resolve upon discontinuation of the drugs, plus supportive therapy (18). Although the dabrafenib and trametinib achieved significant efficacy, the side effects that resulted were also obvious. Combined with the results of the study of 2 patients, we can conclude that dabrafenib and trametinib offers more advantages than disadvantages in the treatment of advanced thyroid carcinoma, and our treatment option is correct and certainly a new attempt for patients with advanced thyroid cancer. In the future, we may need to set up larger clinical trials to add evidence of the feasibility of this option.

## Conclusion

We report 2 rare cases of advanced thyroid carcinoma and point out the effectiveness of the combination of the 2 targeted drugs. The conclusion that dabrafenib and trametinib contributes to the management of advanced thyroid carcinoma remains to be further confirmed. We hope that our treatment procedures and initial outcomes can serve as a reference for the diagnosis and treatment of similar patients.

## Data availability statement

The original contributions presented in the study are included in the article/**Supplementary material**. Further inquiries can be directed to the corresponding authors.

## Ethics statement

The studies involving human participants were reviewed and approved by ethics committee of the West China Hospital, Sichuan University. The patients/participants provided their written informed consent to participate in this study. Written informed consent was obtained from the individual(s) for the publication of any potentially identifiable images or data included in this article.

## Author contributions

XP managed the cases, collected patient data and images, and wrote the manuscript. JL, ZL and KZ conducted the study and revised the manuscript. All authors contributed to the article and approved the submitted version.
