# Supplementary figures and images for: Case report: Visibly curative effect of dabrafenib and trametinib on advanced thyroid carcinoma in 2 patients
Source: Front Oncol. 2023 Jan 5;12:1099268. doi: 10.3389/fonc.2022.1099268 (PMC9850096; doi:10.3389/fonc.2022.1099268)

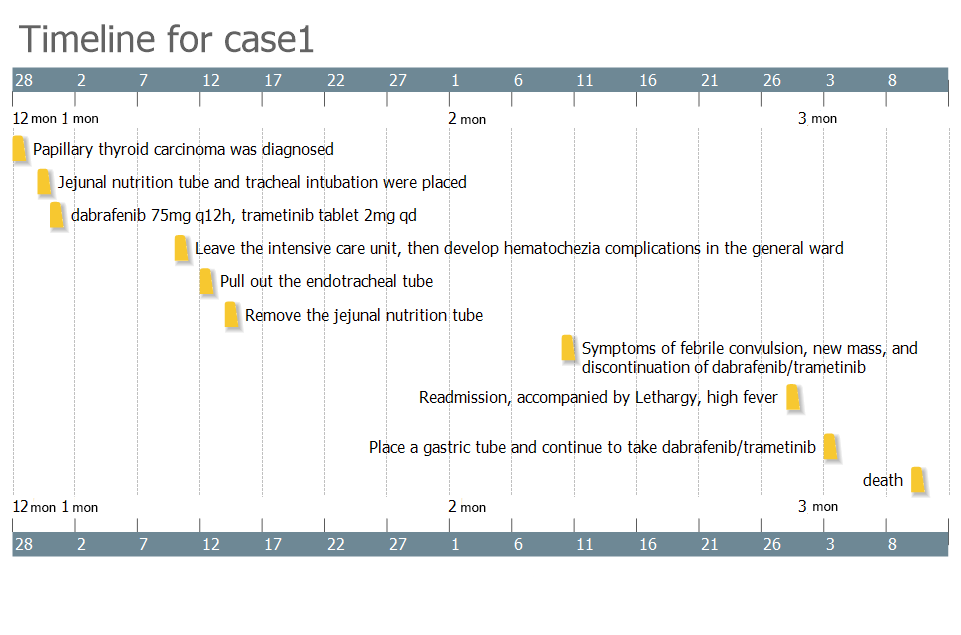

Supplement: Supplementary file 1 [file Image_1.tif]

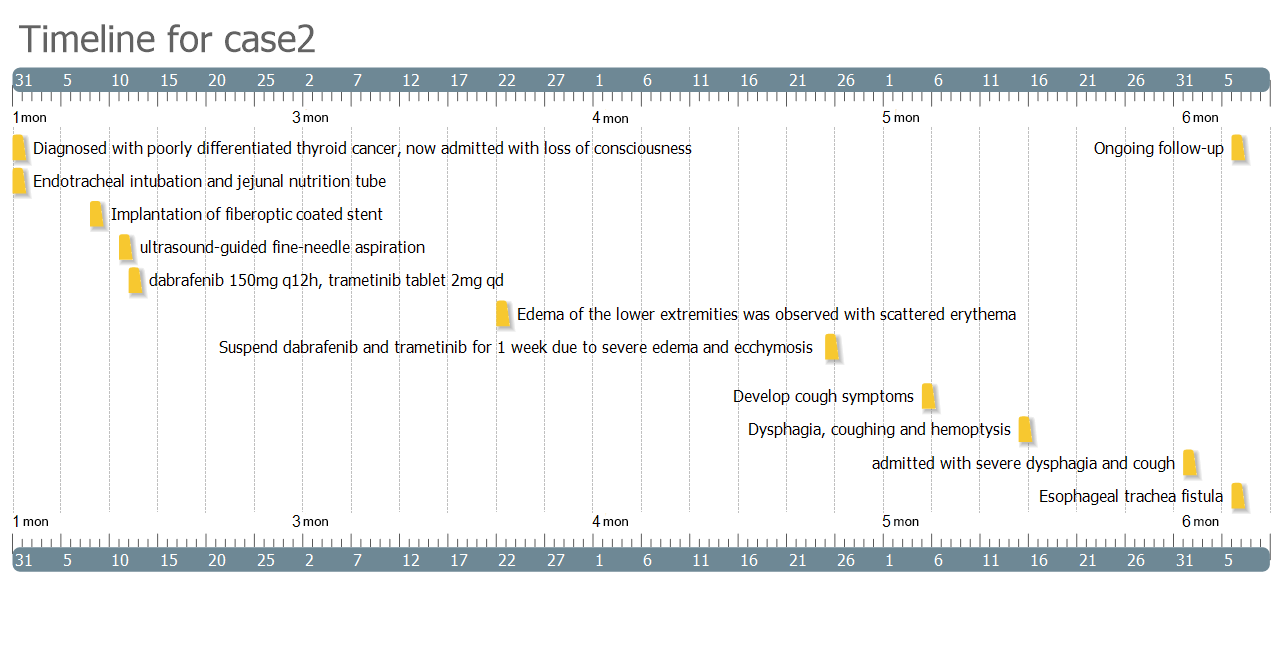

Supplement: Supplementary file 2 [file Image_2.tif]
